# Supplementary material for: Patterns of Intron Gain and Loss in Fungi
Source: PLoS Biol. 2004 Nov 30;2(12):e422. doi: 10.1371/journal.pbio.0020422 (PMC532390; doi:10.1371/journal.pbio.0020422)
Supplement: Table S1 — Also available at http://genes.mit.edu/NielsenEtAl/. (4.3 MB ZIP). [file pbio.0020422.st001.zip › NielsenEtAl/html/926.html]

AN4430.1.NCU01666.1.MG01104.1.FG06282.1


```
 CLUSTAL W (1.82) Multiple Sequence Alignments - Introns Inserted


Sequence 1: NCU01666.1	330 aa
Sequence 2: FG06282.1	312 aa
Sequence 3: MG01104.1	316 aa
Sequence 4: AN4430.1	326 aa
Alignment Length: 333 aa
Number Identitical Residues: 199 aa
Alignment Score (without introns) 8112


MG01104.1 	MAS---------RSLLSSTWRAATAAA-LR--PTAAVRHSS-SSSTSAIAYKALRRRQ-A
NCU01666.1	MASRGLVMSAPLRRISAKARDAAPVAR-QSLPAAIGVRYSSHSSSTSAIAYKAIRRRH-A
FG06282.1 	MAS--------LRSLTASLRWVAPAR------GVAAVRYS--SSSTSAIAYKALRRRS-A
AN4430.1  	MAFR--YTRMLSKTASSSFLSAASRTSGLATRSTFATTTRASSSSTSALAYKALHRRSPL
          	**          :   :.   .*.   .     . ..   : ******:****::** . 

MG01104.1 	PLPTSDSPPAWSSAQAAVSNILYETPTPSTAPPKRHILNCLVQNEPGVLSRVSGILAARG
NCU01666.1	PLPAADAPPAWSSAQAAVSNILYETPSPSLAPPKRHILNCLVQNEPGVLSRVSGILAARG
FG06282.1 	PLPVRDNPPAWS-AQSAVSNILYETPVPSTVPPKRHILNCLVQNEPGVLSRVSGILAARG
AN4430.1  	TLPVTDVSQQWD-AQTAVSSILYETPVRPTNPPKRHVLNCLVQNEPGVLSRVSGILAARG
          	.**. * .  *. **:***.******  .  *****:***********************

MG01104.1 	FNIDSLVVCSTEVADLSRMTIVLTGQDGVVEQARRQLEDLVPVWAVLDYTDSPLVQRELL
NCU01666.1	FNIDSLVVCNTEVDDLSRMTIVLTGQDGVVEQARRQLEDLVPVWAVLDYSKAALVQRELL
FG06282.1 	FNIDSLVVCNTEVEDLSRMTIVLTGQDGVVEQARRQLEDLVPVWAVLDYTAAALVQRELL
AN4430.1  	FNIDSLVVCNTEVEDLSRMTIVLQGQDGVVEQARRQLDDLVPVWAVLDYTDSALVQRELL
          	*********.*** ********* *************:***********: :.*******

MG01104.1 	LAKVNILGQEYFEELLDHHREITSADAEVLA---SSEGEQTLEQVAADFHPSRLAASEAL
NCU01666.1	LAKINILGPEYFEELLAHHREITAEATEGESGKLENGGEHSLEETAKDFHPSRLVASEAL
FG06282.1 	LAKINILGPEYFEELLNHHREIAAGEYDVDN--AHEAIERSISDTAKDFHPSKLAVSQAL
AN4430.1  	LAKVSILGPEYFEELLQHHREITTPVESMEAQ--KKSTDSKASTKTEEYHPRNLPSSQAL
          	***:.*** ******* *****::   .       .  : . .  : ::** .*  *:**

MG01104.1 	RHKHEHLKSITYFTHQFGGKVLDISTNSCIVEL2SAKPSRIDSFLKLIGPFGILESARTG
NCU01666.1	RHKHEHLKSITYFTHQFGGKVLDISTNSCIVEV2SAKPVRIDSFLKLIAPFGILESARTG
FG06282.1 	RHKHEHLKSITYFAHQFGGKVLDISNSSCIVEV2SAKQSRIDSFLKLVAPFGILESARTG
AN4430.1  	RHKHEHLDAITRLTHQFGGKVLDISTNNCIVEL2SAKPSRIDSFMKLISPFGILESTRTG
          	*******.:** ::***********...****: ***  *****:**:.*******:***

MG01104.1 	LMALPRSPLYGPNEEEALIKEADDIVDASQLPPG
NCU01666.1	LMALPRSPLYEHG-EETHVKEADDVVDASQLPPG
FG06282.1 	LMALPRSPLNEAS-QEAVTMEADDVVDASQLPPG
AN4430.1  	LMALPRSPLFEAN--EELEKEAADVVDASTLPPG
          	*********   .  *    ** *:**** ****
```
